# Strategies to overcome CAR-T cell resistance in clinical work: A single-institute experience

**DOI:** 10.3389/fimmu.2022.929221

**Published:** 2022-08-10

**Authors:** Feifei Nan, Xiaorui Fu, Xinfeng Chen, Ling Li, Xin Li, Jingjing Wu, Xiaoyan Feng, Xiaolong Wu, Jiaqin Yan, Mingzhi Zhang

**Affiliations:** Department of Oncology, The First Affiliated Hospital of Zhengzhou University, Zhengzhou, China

**Keywords:** CAR-T cell therapy, resistance, PD-1 inhibitor, ibrutinib, venetoclax

## Abstract

The emergence of chimeric antigen receptor (CAR) T cell therapy has shifted the paradigm of malignant tumor treatment, especially the advent of CD19-directed CAR-T cell therapy for the treatment of relapsed/refractory (R/R) B-cell malignancies. Although CAR-T cell therapy has promising effects, some patients are resistant to this treatment, leaving them with limited options. Therefore, strategies to overcome resistance to CAR-T cell therapy are needed. We retrospectively studied three R/R diffuse large B-cell lymphoma patients who were resistant to CAR-T cell therapy and whose disease was controlled after receiving pembrolizumab, 21D4 CAR-T cells, or ibrutinib and venetoclax. Some promising prevention and treatment strategies to overcome treatment resistance are also discussed.

## Introduction

Chimeric antigen receptor (CAR)- T cell therapy is a novel antitumor therapy, especially for lymphohematopoietic malignancies, and provides opportunities for cure for patients who have received multiple lines of treatment. CD19 CAR-T cells are typically used as treatment. The complete remission (CR) rate for B-cell malignancies is 45–90% with CD19-directed CAR-T cells (1–8). However, some patients do not respond to CD19 CAR-T cell therapy or eventually experience relapse. Therefore, more attention should be given to this group of patients. The mechanisms that underlie relapse are complex and varied, including cancer cell, CAR-T cell, and microenvironment factors (9), and corresponding countermeasures for resistance that develops due to different machanisms are being clinically explored. We herein summarize the experiences of our center in overcoming resistance to CAR-T cell therapy in clinical work and review the mechanisms of and methods for solving this problem in B-cell malignancies.

## Patients and methods

### Patients

We retrospectively reviewed data for three patients treated at the First Affiliated Hospital of Zhengzhou University between July 2017 and June 2019. All patients had relapsed or refractory (R/R) B-cell lymphoma. Before CAR-T cell therapy, all patients received more than two lines of treatment, including rituximab. Two patients had central nervous system (CNS) invasion. One patient received high-dose chemotherapy combined with autologous stem cell transplantation. The patients provided written informed consent according to the Declaration of Helsinki and in accordance with the guidelines presented at the International Conference on Harmonization Guidelines for Good Clinical Practice. All relevant ethical regulations were followed in this study.

### CAR-T cell generation and infusion

Peripheral blood mononuclear cells (PBMCs) were collected from the patients, and CD3+ T cells were isolated from PBMCs. After 3 to 4 weeks of preparation, CAR-T cells were collected and frozen. The patients received 3 days of fludarabine and cyclophosphamide treatment for lymphodepletion and then infusion of CD19 CAR-T cells. Changes in routine blood, liver, and kidney functions, electrolytes, cytokines, and other indicators were closely monitored.

### Follow-up

Response to treatment was evaluated by PET-CT, CT, or MRI every month for the first 3 months and then every 3 months for the first year. Bone marrow and cerebrospinal fluid (CSF) were evaluated at 3 weeks after the initial infusion. The Lugano Revised Criteria were used for the assessment of response [complete response (CR), partial response (PR), stable disease (SD), and progressive disease (PD)]. The cutoff date was April 11, 2022.

## Results

**Figure 1:**
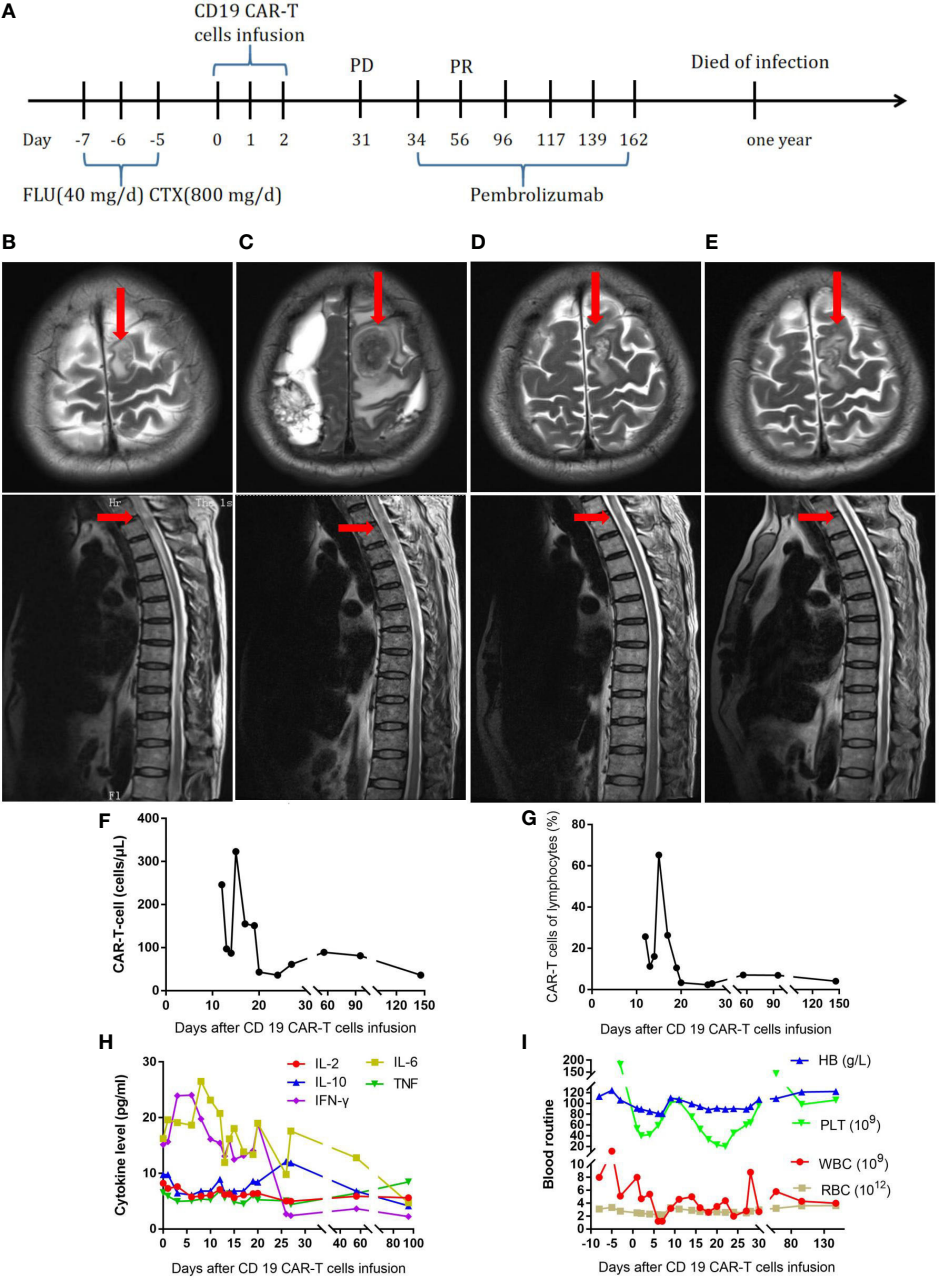
**(A)** Timeline of the treatment process. **(B)** MRI before CAR-T-cell therapy. **(C)** MRI thirty-one days after CAR-T-cell therapy. **(D)** MRI after two doses of pembrolizumab. **(E)**, MRI ten months after CAR-T-cell therapy. **(F)**, Resolution of CAR-T-cell expansion after CAR-T-cell infusion. **(G)**, Percentage of CAR-T cells in lymphocytes after CAR-T-cell infusion. **(H)**, Cytokine levels after CAR-T-cell infusion. **(I)**, Routine blood examination before and after CAR-T-cell infusion.

The first patient was a 59-year-old man (**Table 1**). In July 2017, the patient developed fever and recurrent nosebleeds. Bone marrow tissue biopsy was performed, showing CD20 (+), CD79a (+), CD3 (–), CD5 (-), CyclinD1 (-), Bcl-2 (+), Bcl-6 (-), CD10 (-), MUM-1 (+), and Ki67 (+>50%), and a pathological diagnosis of high-grade B-cell non-Hodgkin lymphoma was made. The PET-CT scan showed multiple dense soft tissue masses in many bones throughout his body, spleen, and bilateral adrenal glands with abnormally increased metabolism (SUVmax: 7.3 for the bilateral adrenal glands). Therefore, this patient was diagnosed as follows: high-grade B-cell lymphoma, NOS stage IVEB, International Prognostic Index (IPI) score 3, and medium to high risk. The patient received six cycles of dose-adjusted (DA)-EPOCH-R (etoposide, 50 mg/m^2^ D1–D4; vincristine, 0.4 mg/m^2^ D1–D4; doxorubicin, 10 mg/m^2^ D1–D4; cyclophosphamide, 750 mg/m^2^ D5; prednisone, 60 mg/m^2^ D1–D5; and rituximab, 375 mg/m^2^ D0) and intrathecal methotrexate + cytarabine. After chemotherapy, the patient was evaluated to have CR. At 4 months later, the patient developed neck pain with limited movement. The MRI result showed abnormal signals in the left frontal lobe and spinal cord at the T2 level, and malignant lymphocytes were found in the CSF, an indicator of progression according to the patient’s history. Subsequently, the patient received one cycle of formustine—100 mg/m^2^ D1, temozolomide—150 mg/m^2^ D1–D5, dexamethasone—40 mg D1–D5, and intrathecal methotrexate + cytarabine. However, his symptoms continued to worsen, with loss of sensory and motor function in the lower extremities (**Figure 1B**). For further treatment, fludarabine (40 mg/day) and cyclophosphamide (800 mg/day) were administered for 3 days to deplete lymphocytes. In total, 2.18 × 10^7^ CAR-T cells were infused into the patient. The treatment process is shown in **Figure 1A**. At 15 days later, the CAR-T cells had expanded to a peak of 303/µl (**Figure 1F**). On the 8th day after the infusion, the patient developed grade 2 cytokine release syndrome (CRS) with fever and anoxia. However, no obvious neurotoxicity was observed. The cytokine levels were not markedly elevated (**Figure 1H**). However, his symptoms continued to worsen. On day 31 after the infusion, the MRI showed enlarged lesions (**Figure 1C**), and the CAR-T cell concentration had decreased to 61/µl (**Figure 1F**). The changes in blood routine were showed in **Figure 1I**. To improve the efficacy, the patient received pembrolizumab (2 mg/kg every 21 days) six times. After two doses, the CAR-T cell concentration increased again to 81/µl (**Figure 1F, G**), and the MRI showed shrinkage of the lesion in the left frontal lobe and spinal cord at the T2 level (**Figure 1D**). No malignant lymphocytes were found in his CSF. The sensory function of the patient was restored, and the motor function in the lower extremities was slightly recovered. The patient’s clinical response persisted for 10 months **Figure 1E**; unfortunately, the patient died of infection.

**Table 1:** Clinical information of the three patients with R/R B- cell lymphoma.

Number	Sex	Age	Diagnosis	Stage	IPI	CNS invasion	Number of prior therapies	Therapies after resistance to CAR-T	Dosage of CAR-T cells
1	Male	59	High-grade B-cell lymphoma, NOS	IVEB	3	Yes	2	Pembrolizumab 2 mg/kg every 21 days	2.18 × 10^7^
2	Female	24	High-grade, double-hit B-cell lymphoma	IVEA	3	Yes	3	21D4 CAR-T cells	2.2 × 10^7^
3	Male	53	Diffuse large B-cell lymphoma, non-GCB	I	0	No	3	Ibrutinib, venetoclax	1 × 10^8^

**Figure 2:**
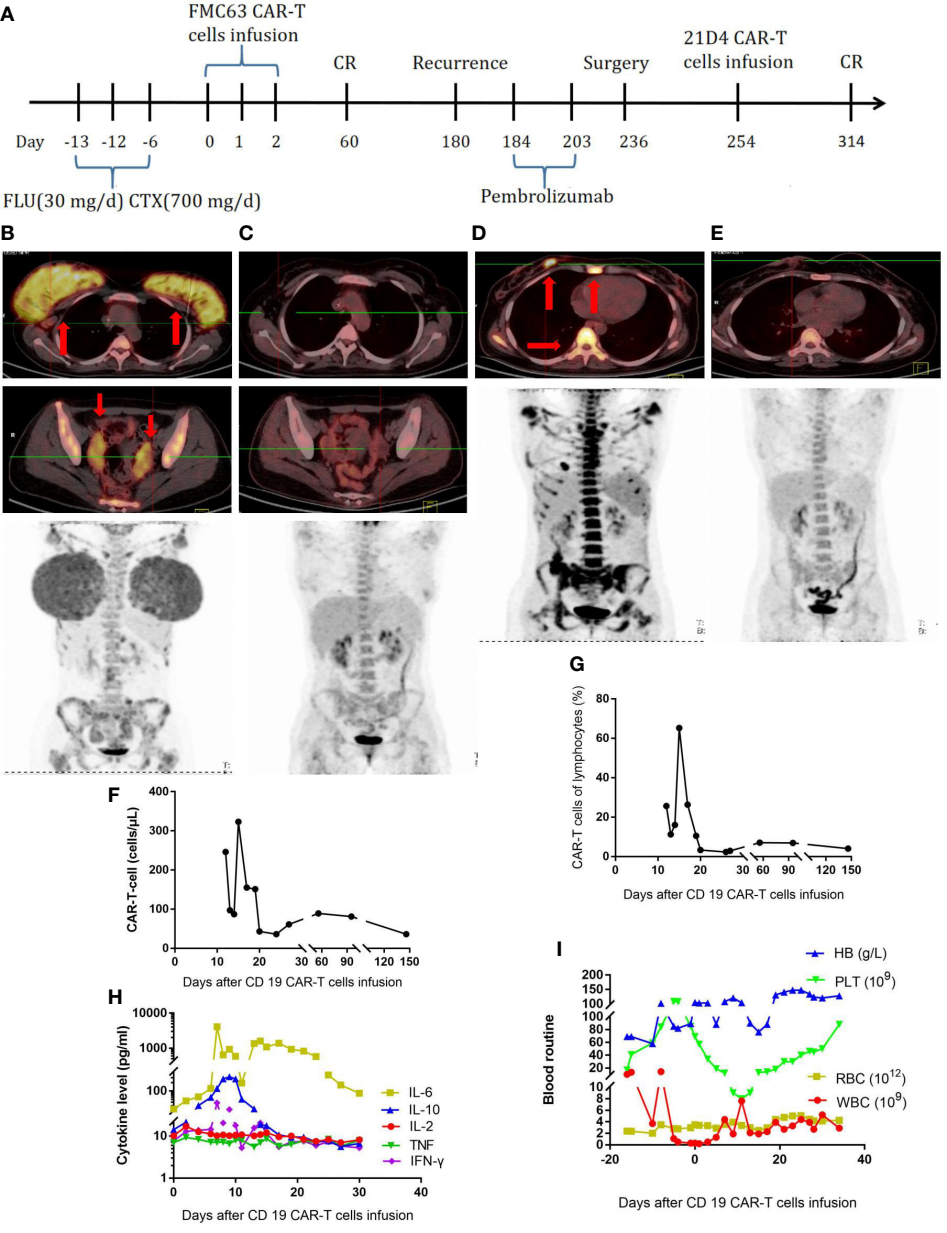
**(A)**, Timeline of the treatment process. **(B)**, PET-CT before the first CAR-T-cell treatment. **(C)**, PET-CT two months after the first CAR-T-cell treatment. **(D)**, PET-CT at the half a year after the first CAR-T-cell treatment and relapse. **(E)**, PET-CT two months after the second CAR-T-cell treatment. **(F)**, CAR-T-cell expansion after CAR-T-cell infusion. **(G)**, Percentage of CAR-T cells in lymphocytes after CAR-T-cell infusion. **(H)**, Cytokine levels after CAR-T-cell infusion. **(I)**, Routine blood examination.

The second patient was a 24-year-old female patient with a diagnosis of high-grade double-hit, stage IVEA B-cell lymphoma (**Table 1**). The PET-CT scan showed invasion of the bilateral mammary glands, left lobe of the liver, cervix and bilateral appendages, bilateral axillary and retroperitoneal lymph nodes, multiple bones throughout the body, and multiple parts of the scalp, spleen, and CNS. A bone marrow smear showed lymphoma involvement. A biopsy of the right mammary gland tumor was performed, showing CD20 (+), CD21 (-), bcl-2 (+), CD10 (+), CD30(-), bcl-6 (+), MUM-1 (+), C-myc (70%+), CD19 (+), TDT (-), MPO (-), Ki-67 (+90%), MYC, and BLC2 rearrangements; a pathological diagnosis of high-grade, double-hit B-cell lymphoma was made. The IPI score was 3, indicating medium to high risk. The patient received one cycle of R-CHOP (rituximab, 750 mg/m^2^ D0; cyclophosphamide, 375 mg/m^2^; vincristine, 1.4 mg/m^2^ D1; epirubicin, 60 mg/m^2^ D1; and prednisone, 60 mg/m^2^ D1–D5), one cycle of hyper-CVAD A (cyclophosphamide, 300 mg/m^2^ Q12H D1–3; vincristine, 1.5 mg/m^2^ D4, 11; epirubicin, 60 mg/m^2^ D4; and dexamethasone, 40 mg D1–D4, D11–14), one cycle of MA (methotrexate—2.5 g/m^2^ D1 and cytarabine—100 mg/m^2^ Q12H D2–3), and two cycles of modified IVAC (ifosfamide, 1 g/m^2^ D1–D5; etoposide, 60 mg/m^2^ D1–5; and cytarabine 1 g/m^2^ Q12H D1–2). She also received intrathecal injections of methotrexate, cytarabine, and dexamethasone twice per cycle. However, her disease was not effectively controlled (**Figure 2B**), and bone marrow invasion developed. On January 26, 2018, the FC (fludarabine—30 mg/day and cyclophosphamide—0.7 g/day) regimen was administered. In total, 2.2 × 10^7^ CAR-T cells were infused. The treatment process is shown in **Figure 2A**. The CD19 CAR-T cells comprising a single-chain fragment variable (scFV) derived from clone FMC63 were generated as previously described (10). At 14 days later, the CAR-T cells had expanded to a peak concentration of 150/µl (**Figure 2F, G**). From the third day, the patient developed fever. On the seventh day, the serum level of IL-6 was significantly elevated (**Figure 2H**), and the patient suffered grade 4 CRS with fever and anoxia. However, no obvious neurotoxicity was observed. On the 17th day, the patient achieved bone marrow minimal residual disease negativity. The changes in blood routine were showed in **Figure 2I**. At 2 months later, the PET-CT scan showed that the patient was in complete remission (**Figure 2C**). Half a year later, a new lesion in the right breast and new lesions in multiple bones throughout the body were detected by PET-CT (**Figure 2D**). A puncture biopsy of the right mammary gland tumor was performed again, and it showed CD20 (+), CD79a (+), CD3 (-), CD19 (100%+), PD-1 (-), PD-L1 (-), and Ki-67 (70%+), followed by a pathological diagnosis of high-grade B-cell lymphoma. Then, the patient received pembrolizumab at 2 mg/kg every 21 days. After two doses of pembrolizumab, no disease remission was achieved. Subsequently, the patient underwent excision of the right breast tumor. The pathological diagnosis was still high-grade B-cell lymphoma, with CD20 (+), CD79a (+), CD3 (-), and Ki-67 (80%+). A p.163 (exon 3) R>L site mutation in CD19 was found by whole-exon sequencing. Therefore, we constructed CD19 CARs comprising scFV derived from clone 21D4 and obtained new CAR-T cells. The 21D4 CAR-T cells were infused. At 2 months later, the PET-CT scan showed that all the lesions had disappeared, and sustained complete remission was achieved (**Figure 2E**).

**Figure 3:**
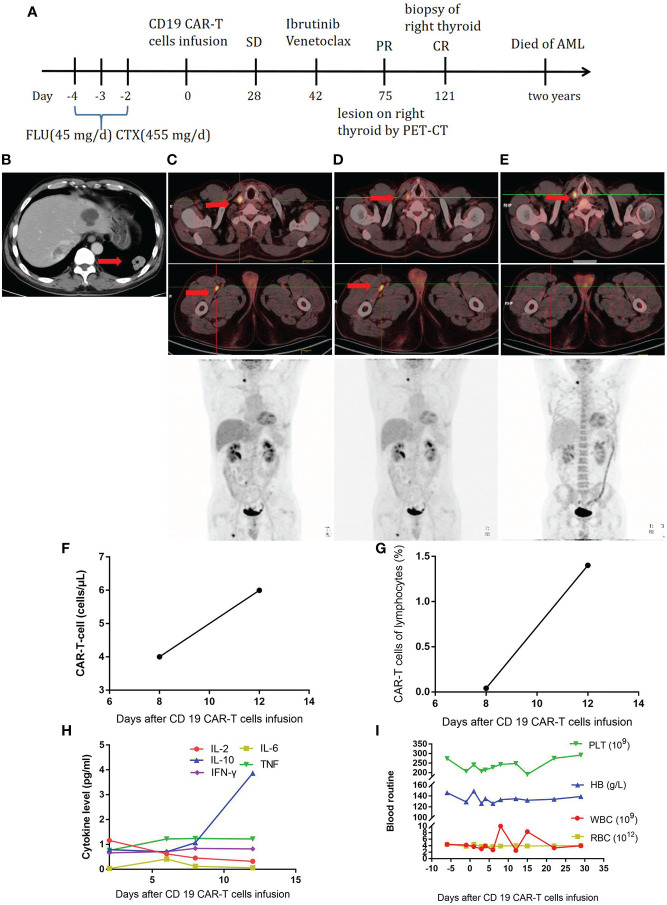
**(A)**, Timeline of the treatment process. **(B)**, CT at relapse after autologous stem cell transplantation. **(C),** PET-CT before CAR-T-cell therapy. **(D)**, PET-CT twenty-eight days after CAR-T-cell therapy. **(E)**, PET-CT one and half months after ibrutinib and venetoclax treatment. **(F)**, CAR-T-cell expansion after CAR-T-cell infusion. **(G)**, Percentage of CAR-T cells in lymphocytes after CAR-T-cell infusion. **(H)**, Cytokine levels after CAR-T-cell infusion. **(I)**, Routine blood examination before and after CAR-T-cell infusion.

The third patient was a 53-year-old male patient (**Table 1**). He developed throat discomfort and hoarseness in May 2018. The CT result showed lesions occupying the anterior wall and laryngopharynx left wall and bilateral cervical lymphadenopathy. A biopsy of the tumor on the left side of the tongue root was performed, showing CD3 (-), CD20 (+), CD10 (-), Bcl-6 (+), MUM-1 (+), Bcl-2 (50%+), c-Myc (60%+), CD5 (-), CyclinD1 (-), CD56 (-), TIA-1 (-), and Ki-67 (90%+); a pathological diagnosis of diffuse large B-cell lymphoma (DLBCL), non-germinal center B-cell-like (GCB) was made. Therefore, the diagnosis was as follows: non-GCB stage I DLBCL, IPI score 0, and low risk. After two cycles of CHOP, the patient’s disease progressed. Then, he received decitabine—10 mg D1–D5, rituximab—375 mg/m^2^ D6, etoposide—60 mg/m^2^ D7–11, arabinoside—100 mg/m^2^ D7–11, and dexamethasone—20 mg D7–11 for four cycles, after which he was evaluated to have CR. After high-dose chemotherapy, he received autologous stem cell transplantation on September 28, 2018. At 8 months later, a lesion was found in the lower lobe of the left lung (**Figure 3B**), and the patient underwent left lower lobectomy. The pathological diagnosis was still DLBCL with CD20 (+), CD79a (+), CD10 (-), Bcl-2 (80%+), and CD19 (100%+). Further testing showed invasion of the right thyroid and proximal segment of the right thigh (**Figure 3C**). Subsequently, the FC regimen was used to deplete lymphocytes. In total, 1 × 10^8^ CAR-T cells were infused. The treatment process is shown in **Figure 3A**. At 28 days later, no remission was observed by PET-CT (**Figure 3D**). The CAR-T cell concentration and cytokine levels were not markedly elevated (**Figures 3F–H**), and no obvious CRS was observed. The changes in blood routine were showed in **Figure 3I**. To improve CAR-T cell efficacy, ibrutinib—420 mg QD and venetoclax—200 mg QD were given to the patient. At 1.5 months later, the PET-CT scan showed that the lesion in the proximal segment of the right thigh disappeared, but there was no change in the right thyroid lesion (**Figure 3E**). A puncture biopsy was performed, and the pathological diagnosis was nodular goiter. Therefore, the patient was evaluated to have CR. Unfortunately, the patient developed acute myeloid leukemia 2 years later.

## Discussion

In the past two decades, CAR-T cell therapy has revolutionized the treatment of hematologic malignancies, especially B-cell lymphoma. Numerous clinical studies have led to great achievements in B-cell lymphoma treatment, which are gradually changing the standard treatment strategy (3, 11–13). However, it should be noted that some patients are resistant to CAR-T cell therapy. Resistance mechanisms can involve CAR-T cell, tumor microenvironment, and/or cancer cell factors (9). Here we report three R/R B-cell lymphoma patients who were resistant to CAR-T cell therapy and the methods we used to reverse resistance.

For the first patient, the CAR-T cells expanded after infusion. However, the patient’s symptoms were not relieved, indicating resistance to CAR-T cell therapy. After two doses of pembrolizumab, a PD-1 inhibitor, the CAR-T cells expanded again. More importantly, the lesions in the CNS were effectively controlled and gradually shrunk with the application of pembrolizumab. The patient’s symptoms were also relieved. All the above-mentioned results suggest that PD-1 inhibitors can synergistically increase the function of CAR-T cells. In a retrospective study, pembrolizumab not only reversed T cell exhaustion but also increased CAR-T cell activation and proliferation, with four of 12 patients achieving clinical responses (14). In another study, three of five patients who were resistant to CD19/20 CAR-T cell therapy achieved an objective response after receiving sintilimab or camrelizumab. In the responders, T cells were activated, but no increase in CAR-T cell concentration was found. The effect of PD-1 blockade has been associated with the expression of PD-L1 in tumor cells and that of PD-1 in tumor-infiltrating T cells (15). In our study, after the application of pembrolizumab in the first patient, the CAR-T cell concentration increased, but this pattern was not seen in the second patient. The second patient did not show PD-1 or PD-L1 expression. Due to the unique location of the lesion, no further pathological biopsy was performed for the first patient, and we did not assess the expression of PD-1 and PD-L1. However, previous studies have suggested that PD-1 and PD-L1 are expressed in a remarkable number of CNS lymph node cells and infiltrating lymphocytes (16, 17). Therefore, we considered that the re-expansion of CAR-T cells and the curative effect were associated with the expression of PD-1 or PD-L1. In addition to the use of PD-1 inhibitors, some researchers have used other methods to inhibit the expression of PD-1 or other checkpoint receptors on CAR-T cells, such as adenine base editors or short hairpin RNAs, and these new CAR-T cells have shown better anticancer effects *in vitro* (18, 19). Nevertheless, further clinical trials should be conducted to determine the effect of such CAR-T cells in patients. Another study showed that blocking PD-1/PD-L1 signaling with an anti-PD-1 nanobody in CAR-T cells reduces survival and diminishes the cytotoxicity of the cells despite increased activation (20). Therefore, more exploration of the influence of checkpoint inhibitors on CAR-T cells should be performed. The characteristics of patients who can benefit from treatment with immune checkpoint inhibitors after failure of CAR-T cell therapy should also be confirmed by more research.

Antigen loss under therapy pressure is considered a mechanism by which tumor cells escape recognition by CD19 CAR-T cells (21). The main reasons for antigen loss are gene mutation, alternative splicing, and epigenetic modification. Exon 2 is essential for the integrity of the CD19-epitope on B-cells, and exons 5 and 6 encode the transmembrane domain (22–24). Mutations of exons 2–5 play an important role in resistance to CD19 CAR-T cells. Oralando and colleagues found that mutations in CD19 exons 2–5 lead to a truncated protein associated with resistance to anti-CD19 CAR-T cell therapy (25). Frameshift mutations of CD19 exon 2 related to intron 2 are also associated with CD19 CAR-T cell resistance (26). For patients with these mutations, targeting a different binding epitope on CD19 may overcome resistance. For our second patient, whose lymphoma relapsed after the first infusion of CAR-T cells, the subsequent pathological examination showed CD19 positivity. Further assessment revealed the presence of an exon 3 site mutation of CD19 (p.163 R>L), and cells with this mutation exhibited resistance to FMC63 CAR-T cells. However, this resistance was effectively eradicated by the use of 21D4 CAR-T cells, which target a different binding epitope than FMC63 CAR-T cells. A further study suggested that 21D4 CAR-T cells exert antitumor activity against B-cell lymphoma with CD19 exon 1 and 2 mutations, which are associated with resistance to FMC63 CAR-T cells (10). In another study, patients with R/R B-cell acute lymphoblastic leukemia (B-ALL) received CNCT19 cells, a new CD19 CAR-T cell produce that targets a different binding epitope on CD19 than FMC63 CAR-T cells. Ninety percent of these patients achieved CR within 28 days, and the median overall survival and progression-free survival were 12.91 and 6.93 months, respectively (27). Alternative splicing is another reason for the loss of CD19 antigen. Alternative splicing leads to the production of alternative mRNA transcripts that encode structurally and functionally different protein isoforms, which affect target recognition by CAR-T cells, resulting in disease recurrence (23, 24, 28). This mechanism may explain the relapse of some patients. In some B-ALL patients, alternative splicing of exon 2 eliminates full-length CD19 and the expression of exon 2 isoform, resulting in a protein that is not recognized by CD19 CAR-T cells and does not activate them (23, 24). The third reason for antigen loss is epigenetic modification, including DNA methylation and histone acetylation, which regulates gene expression without changing the DNA sequences. DNA promoter hypermethylation leads to a lack of CD19 expression, which is associated with resistance of chronic lymphocytic leukemia (CLL) to CD19 CAR-T cell therapy, and this situation can be reversed by treatment with a demethylating agent (29). In addition, epigenetic modifiers enhance the antitumor ability of CAR-T cells by increasing the antigen expression on the surface of B-lymphoma cells. The histone deacetylase inhibitor (HDACi) chidamide promotes the antitumor ability of CD22 CAR-T cells by affecting CD22 distribution (30). Pretreatment with HDACis before CD20 CAR-T cell therapy enhances cytotoxic activity by inducing the acetylation of H3K9 in the CD20 promoter in a mouse cell model of Burkitt’s lymphoma (31). Agents such as 5-aza-2’-deoxycytidine increase the expression of the antigen CD19 on the surface of lymphoma cells, and patients with B-cell lymphoma are more susceptible to CD19 CAR-T cells (32). Although we did not use epigenetic modifiers in the three patients, we think that they are promising for improving the effect of CAR-T cell therapy.

The third patient showed no response to CD19 CAR-T cells. After treatment with ibrutinib and venetoclax, the patient achieved CR. Ibrutinib was originally used to cure B-cell lymphoma because of its effect as a Bruton’s tyrosine kinase (BTK) inhibitor. However, through the inhibition of interleukin-2-inducible T cell kinase (ITK), ibrutinib can increase CD4+ and CD8+ T cell numbers, especially in effector/effector memory subsets, which is not observed with acalabrutinib (33). Ibrutinib also improves the T cell cytotoxicity against CLL cells induced by anti-CD19/CD3 bispecific antibodies in patients (34). Acalabrutinib also increases T cell function and cytotoxic activity by reducing PD-1 and CTLA-4 expression (33, 34). Additional research has shown that ibrutinib increases the number and activity of T cells in the apheresis product and the persistence of activated T cells (35). Ibrutinib also increases the proportion of memory CAR-T cells by inhibiting terminal cell differentiation (36), improves cytokine release capacity, and reduces the expression of exhaustion markers in CAR-T cells (37). In addition to its direct influence on T cells, ibrutinib inhibits lymphoma cell adhesion and migration by decreasing tissue-homing chemokines such as CXCL-12 and CXCL13 so that malignant B-cells infiltrate the peripheral blood to move from protective niches into the circulation and are then destroyed by circulating CAR-T cells (38–40). In a study with a xenograft mantle cell lymphoma (MCL) model, CTL019 + ibrutinib led to better long-term remission than CTL019 alone (41). Based on the above-mentioned laboratory findings, some clinical trials have explored the role of ibrutinib in patients. Gill SL and colleagues showed that, after at least 6 months of exposure to ibrutinib, patients with CLL achieved beneficial effects from CD19 CAR-T cell therapy. The CR rate was 44% on the third month, and the overall and progression-free survival rates at 4 years were 84 and 70%, respectively (42). In another clinical study, seven patients (MCL and follicular lymphoma) received salvage ibrutinib treatment after the failure of first-line CD19 CAR-T cell therapy. Then, the patients received second-line CD19 CAR-T cell therapy, which resulted in better efficacy and CAR-T cell amplification than the first-line therapy (43). In addition to ibrutinib, we used venetoclax for the third patient. Venetoclax is an effective and selective BCL-2 inhibitor that disrupts BCL-2 signaling and induces apoptosis in hematological malignancies (44). For CLL patients, the expression of BCL-2 on T cells increases the differentiation of regulatory T cells (Tregs), the exhaustion of cytotoxic T lymphocytes (CTLs), and the levels of suppressive cytokines, such as IL-10 and TGF-β (45). After treatment with venetoclax, effector T cells are activated, the cytotoxicity of T cells is enhanced, and Tregs are depleted (46). Venetoclax also augments the cytotoxicity of effector T cells when combined with immunotherapies, such as CTL-based therapies and immune checkpoint inhibitors (47, 48). Especially when combined with CD19 CAR-T cells, venetoclax upregulates the expression of the CD19 antigen in tumor cells, improves the persistence of CAR-T cells, and improves antitumor efficacy (49, 50). Therefore, ibrutinib and venetoclax are promising treatments to enhance the efficacy of CAR-T cells. For the third patient, we did not detect the antitumor ability of CAR-T cells after treatment with ibrutinib and venetoclax because the patient refused to provide blood at the subsequent follow-up. More experimental and clinical studies are needed to confirm whether ibrutinib and venetoclax can work synergistically with CAR-T cells.

In conclusion, the mechanisms of resistance to CAR-T cell therapy are complex and diverse. There have been many attempts to overcome resistance to CAR-T cells. The three cases in this article demonstrate the methods that we are exploring in clinical work. PD-1 and BTK inhibitors are easy to obtain and have shown promising effects in many studies. Gene mutation and alternative splicing are common reasons for loss of the CD19 antigen. Therefore, for patients, whole-exon sequencing is needed to determine the corresponding treatment. Epigenetic modifiers such as decitabine and chidamide can restore the expression of the CD19 antigen in some patients. In addition, many other strategies have been used in studies to circumvent resistance to CAR-T cells, such as PI3K inhibitors, CAR-T cells targeting multiple antigens, and CAR-T cells combined with hematopoietic stem cell transplantation. Once patients show resistance to CAR-T cell therapy, it is important to find an appropriate treatment as soon as possible because the disease will progress rapidly. More systematic studies are needed to identify the individual characteristics of patients that aid in the rapid identification of an appropriate treatment that is effective in overcoming resistance to CAR-T cell therapy.

## Data availability statement

The original contributions presented in the study are included in the article/supplementary material. Further inquiries can be directed to the corresponding author.

## Ethics statement

The studies involving human participants were reviewed and approved by the Ethics Committee of the First Affiliated Hospital of Zhengzhou University. The patients/participants provided their written informed consent to participate in this study. Written informed consent was obtained from the individual(s) for the publication of any potentially identifiable images or data included in this article.

## Author contributions

FN wrote the manuscript. FN and MZ approved the final version of the manuscript and are accountable for all aspects of the work. XFu, XC, LL, XL, XFe, XW, JW, and JY collected and analyzed the data. All authors contributed to the article and approved the submitted version.

## Conflict of interest

The authors declare that the research was conducted in the absence of any commercial or financial relationships that could be construed as a potential conflict of interest.

## Publisher’s note

All claims expressed in this article are solely those of the authors and do not necessarily represent those of their affiliated organizations, or those of the publisher, the editors and the reviewers. Any product that may be evaluated in this article, or claim that may be made by its manufacturer, is not guaranteed or endorsed by the publisher.
